# Is Reformulation Still a Suitable Goal for Sugary Beverage Taxes? A Response to Recent Commentaries

**DOI:** 10.34172/ijhpm.2023.8366

**Published:** 2023-12-13

**Authors:** Hannah Forde, Tarra L. Penney, Martin White, Jean Adams

**Affiliations:** ^1^Nuffield Department of Primary Care Health Sciences, University of Oxford, Oxford, UK; ^2^School of Global Health, York University, Toronto, ON, Canada; ^3^MRC Epidemiology Unit, University of Cambridge, Cambridge, UK

 In 2019, one year after the UK government implemented a sugary beverage tax (SBT) known as the “Soft Drink Industry Levy” (SDIL), we interviewed 18 marketing experts.^1^ We explored how soft drink companies adapt their marketing in response to a SBT, specifically by adjusting products, their placement, promotion, and pricing (the “4Ps”). The framework we developed shows how companies actively assess their context in order to inform decisions about marketing, meaning that: (*i*) company reactions to a SBT could be pre-empted if enough is known about these contextual factors from the outset; and (*ii*) SBTs could be designed to mitigate the potentially health-undermining reactions of market leaders. Since the publication of our original work, further evidence has mounted to show that companies have responded to the threat and implementation of SBTs using costly marketing measures, such as price promotions in Bermuda,^2^ new product development in Barbados,^3^ and additional advertising in South Africa and Washington, the United States.^4,5^ These responses reflect an ongoing process of cost-benefit analysis and adaptation that corresponds with those depicted in our framework.

 Commentaries published about our original work have also enhanced our understanding of marketing responses to SBTs and the remaining gaps in research.^6-14^ More in-depth data, in the form of longitudinal and insider interviewees could improve future research, as would building on an updated, broader view of marketing than the “4Ps” that encompasses a wider range of market and strategic actions.^11^ Together, these approaches would allow for the testing and development of our framework with concepts that did not surface in our interviews.^12,13^

 In our original work, we documented the potential marketing ramifications of SBTs without demarcating those that would improve or worsen public health. Reformulating sugary drinks to low sugar alternatives (“nutrient reformulation”), often through use of non-nutritive sweeteners, is a frequent consequence of SBTs.^15^ For some health-related SBTs including the SDIL,^16^ incentivising industry to reformulate their products is stated as an explicit policy goal, and reinforced by the policy’s design. For the SDIL at least, it seems that goal was achieved for the products of some but not all soft drink companies.^17^ Here, we reflect on the commentaries on our original work in light of new evidence about reformulation, and interpret potential implications for policy-makers, soft drinks companies and researchers.

 As noted by several commentaries,^6,8,9^ understanding and concern about the role of reformulation, and in turn, non-nutritive sweeteners, in healthy diets has evolved since the SDIL was implemented. Firstly, further evidence now documents the health outcomes of non-nutritive sweetener consumption, to the extent that in 2023 the World Health Organization (WHO) cautiously recommended that non-sugar sweeteners should not be used as a means of achieving weight control or reducing the risk of non-communicable diseases (NCDs).^18^ This recommendation primarily reflects evidence of a potential association between the consumption of non-sugar sweeteners and increased risk of NCDs (eg, type 2 diabetes, cardiovascular disease) in the longer term.^18^

 Secondly, food and drink processing – beyond the ingredients, nutrient composition, and energy content of food– is of growing concern.^9,19^ Studies have demonstrated that consuming highly industrially processed (“ultra-processed”) food and drink is associated with increased risk of numerous NCDs.^20^ New evidence from a prospective, multinational cohort study specifically suggests that higher consumption of ultra-processed, sugar-sweetened beverages are associated with risk of cancer and cardiometabolic diseases.^21^ While reformulated soft drinks may reduce the health impacts of soft drink consumption that relate to sugar, it is possible that any health consequences related to ultra-processing will persist — or possibly magnify — following reformulation.

 Finally, as noted by one commentary,^8^ the risk of surrogate marketing — where non-regulated products are used to promote regulated products — undermining reformulation is also currently unknown. Anecdotal evidence of surrogate marketing emerged following the announcement of the SDIL. Coca-Cola publicised their “One Brand” marketing strategy, with planned to make cans of low-sugar Coca-Cola variants (eg, Diet Coke and Coca-Cola Zero) predominantly red, and thus more closely resemble those of regular Coca-Cola.^22^ The change in packaging was purportedly to encourage the sales of low-sugar variants, but some of our interviewees also suggested it may work to perpetuate promotion of the core [high-sugar] Coca-Cola brand.^1^ Comprehensively measuring the extent and nature of surrogate marketing in future research would help to demonstrate whether sales of high-sugar original products are in fact bolstered by the availability of reformulated alternatives, in turn showing whether and how SBTs instigate long term changes in soft drink market composition.

 As the systemic consequences of SBTs are now understood to evolve and develop over time,^23^ it is increasingly important for policy-makers to be clear of longer term policy objectives. While for policies like the SDIL, the short-term objective of nutrient reformulation has reduced sugar consumption by altering the *content *of soft drinks consumed, a greater impact on diet-related health could be achieved if an SBT were designed to reduce the *overall volume* of soft drinks consumed.

 Changing the messaging about a SBT’s mechanism and purpose from the outset could produce several ramifications for policy-makers, companies, and researchers. As soft drink companies’ scan the policy horizon when deciding how to react to SBTs,^1^ a government’s longer term goal of reducing the volume of soft drinks consumed could discourage attempts to sustain sugary drinks sales (eg, increased advertising) and/or nutrient reformulation. Instead, it might encourage companies to invest in alternative, healthier product categories (eg, waters, unsweetened teas, coffees) and instigate “whole food reformulation”^24^: the development of less processed, healthier alternatives. Policy-makers reinforcing an SBT with other measures to reduce the volume of soft drinks produced and sold, for example concurrent marketing restrictions, could incentivise this further. Such complementary policies could be defined by product categories to shift companies away from ultra-processed sugary beverages; potentially building on plans for new advertising policies in the United Kingdom to be defined by both nutrient and category definitions.^25^ Coupling this with incentives for the production and sales of health-promoting foods and drinks (eg, subsidising crops other than sugar beet) could mitigate the potentially regressive consequences of this approach and make it more economically viable for businesses.

 Reformulation may continue to be a core response to SBTs, but recent evidence challenges whether this is the most effective way to maximise the potential health benefits of a SBT. Focusing more on reducing the total volume of soft drinks consumed, and achieving through the integration of a range of policies, may be a better way to develop longer term, sustainable changes that contribute to a healthier commercial food system.

## Acknowledgements

 We extend our gratitude to Dr. Jessica Renzella at the University of Oxford for reviewing an early draft of this work.

## Ethical issues

 Not applicable.

## Competing interests

 Authors are in receipt of additional grants from NIHR (HF, MW, JA), Wellcome Trust (HF), the University of Oxford’s Nuffield Department of Primary Care Health Sciences (HF), BBSRC and ESRC (MW, JA). Authors have received payments to cover travel expenses to the European Association for the Study of Obesity’s Early Career Network Winter School 2022 (HF), and payment and travel expenses for meetings of the UK Scientific Advisory Committee on Nutrition from the UK Government’s Department of Health & Social Care (JA). HF has received an annual research allowance and a termly honoraria as a Holywell Manor Research Associate at the University of Oxford’s Balliol College and as a Junior Research Fellow at the University of Cambridge’s Wolfson College, respectively. MW and JA report payment for organisation and delivery of an educational event from Bloomberg Philanthropies. MW reports membership of the scientific advisory committee of the Food Foundation (UK) and JA reports membership of advisory boards for various projects funded by NIHR. TLP reports no competing interests.

## Funding

 Hannah Forde is currently funded by the COPPER project. This COPPER project is funded by the NIHR Public Health Research programme (NIHR133887). Jean Adams and Martin White are supported by the Medical Research Council [Grant number: MC_UU_00006/7].

## Disclaimer

 The views expressed are those of the author(s) and not necessarily those of the NIHR or the Department of Health and Social Care.
